# Special issue: Artificial intelligence in genomics

**DOI:** 10.1007/s00439-022-02472-7

**Published:** 2022-07-30

**Authors:** Anne-Laure Boulesteix, Marvin Wright

**Affiliations:** 1 Department of Medical Information Processing, Biometry and Epidemiology (IBE), Ludwig-Maximilians-University of Munich, Marchioninistr. 15, 81377 Munich, Germany; 2 Department of Biometry and Data Management, Leibniz Institute for Prevention Research and Epidemiology – BIPS, Achterstraße 30, 28359 Bremen, Germany

Artificial intelligence (AI) is increasingly used to extract knowledge from complex and large data sets such as those generated by high-throughput molecular technologies. AI methods are becoming ubiquitous in various fields related to genetics and genomics, including those that have long been dominated by classical statistical modeling approaches. The new methods provide promising new opportunities but they also come along with a number of methodological difficulties. Both are addressed in this special issue, *Artificial Intelligence in Genomics*. In an ideal world, analyses based on complex algorithms should *handle complexity* appropriately and be *reproducible*, *interpretable*, *automatic*, and *translationable*. The six articles by Manduchi et al., Watson, Musolf et al., Couckhuyt et al., Treppner et al., and Xu & Mansmann give insights into these major challenges and possible solutions.

Couckhuyt et al. provide an overview of the whole process from exploratory data analyses using machine learning to translation into a clinical setting, which they define as “translational machine learning”. They particularly address issues related to the experimental setup, the choice of computational methods, interpretability, and reproducibility while emphasizing the arising challenges. Some of these issues are scrutinized individually in more detail in further articles of the special issue.

Xu and Mansmann provide a reproducibility study devoted to a recent article suggesting a personalized, therapeutic decision support tool for acute myeloid leukemia patients in the field of molecular medicine. In view of the numerous minor and major problems they encounter in their efforts towards reproducing the results, they conclude that making code and data publicly available, although a major necessary step, does not guarantee full reproducibility and transparency of the performed analyses. Based on their experience, they discuss possible ways to improve scientific practice with respect to reproducibility and give recommendations for future reproducibility studies in the form of a checklist.

Among machine learning approaches, deep learning techniques are becoming increasingly popular, especially in applications featuring complex patterns that cannot be captured using standard modeling approaches relying on strong assumptions, e.g. linearity. Treppner et al. provide a gentle introduction to and an overview of deep generative models, which can be used to extract complex information from high-throughput molecular data, such as pathways or gene programs. A particular emphasis is set on methods that enable inference of relationships between the latent variables learned by the models and the observed data for the purpose of interpretability.

More generally, a major issue related to the use of AI methods is their lack of interpretability. While linear regression models have a simple interpretation in terms of linear effects of covariates on the dependent variable, data-driven machine learning models are often perceived as a “black box”. Two papers in this special issue discuss how to improve the interpretability of such models: Watson provides a gentle introduction to interpretable machine learning, discussing limitations of existing approaches and open challenges in a broad context. The review by Musolf et al. focuses on so-called feature importance measures that have been proposed and applied in the context of genetic association studies and related fields. As opposed to black-box algorithms returning only a prediction rule but no clue on how these predictions are derived, the objective of feature importance measures is to provide information on the contributions of the candidate features to the prediction process.

The number of recently developed methodological variants and applications of AI methods in genomics is overwhelming. A crucial question that arises in this context is how to choose a particular algorithm over other (related) ones. An increasingly popular class of methods addressing this issue is automatic machine learning (AutoML), which is outlined at the end of the paper by Musolf et al. and addressed in detail by Manduchi et al., who write “The goal of automated machine learning (AutoML) is to let a computer algorithm identify the right algorithms and hyperparameters thus taking the guesswork out of the optimisation process.” Their paper provides an intuitive introduction into such methods with a focus on applications to genetic analysis of complex traits and omics data. They review major user-friendly autoML tools, the most important processes that can be automatised with such frameworks, and the algorithms they use for the optimisation process.

We would like to thank all authors for their fantastic work and timely submissions in spite of the challenging pandemic situation, as well as the editors, editorial office, and production team for their constant support in the publication of this special issue. This issue will hopefully help bridge the gap between the current thrilling developments in AI and applications in genetics and genomics.

